# The *POLG* Variant c.678G>C; p.(Gln226His) Is Associated with Mitochondrial Abnormalities in Fibroblasts Derived from a Patient Compared to a First-Degree Relative

**DOI:** 10.3390/genes16020198

**Published:** 2025-02-05

**Authors:** Imra Mantey, Felix Langerscheidt, Çağla Çakmak Durmaz, Naomi Baba, Katharina Burghardt, Mert Karakaya, Hans Zempel

**Affiliations:** 1 Institute of Human Genetics, University Hospital Magdeburg, 39120 Magdeburg, Germany; 2 Institute of Human Genetics, Faculty of Medicine and University Hospital Cologne, University of Cologne, 50937 Cologne, Germany; 3 Center for Molecular Medicine Cologne (CMMC), University of Cologne, 50937 Cologne, Germany; 4 Institute of Human Genetics, Jena University Hospital, Friedrich Schiller University, 07747 Jena, Germany

**Keywords:** mitochondriopathy, Alpers-Huttenlocher syndrome, mtDNA copy number, mtDNA depletion, polymerase-gamma, spinal muscular atrophy, rare disease

## Abstract

Background: The nuclear-encoded enzyme polymerase gamma (Pol-γ) is crucial in the replication of the mitochondrial genome (mtDNA), which in turn is vital for mitochondria and hence numerous metabolic processes and energy production in eukaryotic cells. Variants in the *POLG* gene, which encodes the catalytic subunit of Pol-γ, can significantly impair Pol-γ enzyme function. Pol-γ-associated disorders are referred to as POLG-spectrum disorders (POLG-SDs) and are mainly autosomal-recessively inherited. Clinical manifestations include muscle weakness and fatigue, and severe forms of the disease can lead to premature death in infancy, childhood, and early adulthood, often associated with seizures, liver failure, or intractable epilepsy. Here, we analyzed fibroblasts from a compound heterozygous patient with the established pathogenic variant c.2419C>T; p.(Arg807Cys) and a previously undescribed variant c.678G>C; p.(Gln226His) with a clinical manifestation compatible with POLG-SDs, sensory ataxic neuropathy, and infantile muscular atrophy. We conducted a battery of functional studies for Pol-γ and mitochondrial dysfunction on the patient’s fibroblasts, to test whether the novel variant c.678G>C; p.(Gln226His) may be causative in human disease. Aims/Methods: We analyzed skin-derived fibroblasts in comparison to a first-degree relative (the mother of the patient), an asymptomatic carrier harboring only the established c.2419C>T; p.(Arg807Cys) mutation. Assessments of mitochondrial function included measurements of mtDNA content, mRNA levels of mitochondrial genes, mitochondrial mass, and mitochondrial morphology. Case Presentation and Results: A 13-year-old male presented with symptoms starting at three years of age, including muscle weakness and atrophy in the lower extremities and facial muscles, which later extended to the upper limbs, voice, and back muscles, without further progression. The patient also reported fatigue and muscle pain after physical activity, with no sensory deficits. Extensive diagnostic tests such as electromyography, nerve conduction studies, muscle biopsy, and MRI were unremarkable. Exome sequencing revealed that he carried the compound heterozygous variants in *POLG* c.678G>C; p.(Gln226His) and c.2419C>T; p.(Arg807Cys), but no other potential genetic pathogenic causes. In comparison to a first-degree relative (his mother) who only carried the c.2419C>T; p.(Arg807Cys) pathogenic mutation, in vitro analyses revealed a significant reduction in mtDNA content (~50%) and mRNA levels of mtDNA-encoded proteins. Mitochondrial mass was reduced by approximately 20%, and mitochondrial interconnectivity within cells was impaired, as determined by fluorescence microscopy and mitochondrial staining. Conclusions: Our findings suggest that the c.678G>C; p.(Gln226His) variant, in conjunction with the c.2419C>T; p.(Arg807Cys) mutation, may compromise mtDNA replication and mitochondrial function and could result in clinically significant mitochondriopathy. As this study is based on one patient compared to a first-degree relative (but with an identical mitochondrial genome), the pathogenicity of c.678G>C; p.(Gln226His) of *POLG* should be confirmed in future studies, in particular, in conjunction with other *POLG*-variants.

## 1. Introduction

Mitochondria are essential cellular organelles for eukaryotes. They supply most eukaryotic cells with the majority of adenosine triphosphate (ATP), which is produced as part of oxidative phosphorylation (OXPHOS) by the protein complexes of the electron transport chain (ETC) at the inner mitochondrial membrane [1,2]. In addition, mitochondria are involved in a variety of crucial cellular mechanisms such as divers metabolisms, apoptosis, endoplasmic reticulum stress response, and the generation of reactive oxygen species (ROS) [2]. Accordingly, mitochondrial homeostasis is crucial in safeguarding mitochondrial function [3], and mitochondrial fusion and fission are important processes in providing it [4].

Mitochondria have their own genome, or their own set of DNA, here referred to as mitochondrial DNA (mtDNA), which is inherited maternally [5]. The double-stranded negatively supercoiled circular genome comprises 16,569 base pairs (bp) and encodes a total of 37 genes (13 proteins essential to the ETC, 22 transfer RNAs (tRNAs), and 2 mitochondrial ribosomal RNAs (rRNAs)) [6,7]. The heterotrimeric enzyme DNA Polymerase-γ (Pol-γ), whose catalytic subunit Pol-γA is encoded by the nuclear gene *POLG*, is the only polymerase responsible for the replication of mtDNA in mammals. The loss or reduced functionality of Pol-γ directly affects mitochondrial DNA synthesis and leads to mtDNA depletion and severe mtDNA deletions [8]. While mtDNA depletion (a reduced copy number of mtDNA) often occurs in diseases with an early onset, mtDNA deletions (a loss of segments of the mtDNA) occur more often in late-onset diseases [9].

Mutations in *POLG* cause a spectrum of disorders characterized by mtDNA instability as mtDNA replication is impaired [10]. This causes mitochondrial dysfunction and neurodegenerative disorders [11,12], which are among the most common causes of inherited mitochondriopathies in children and adults, referred to as POLG-spectrum disorders (POLG-SDs) [12,13]. This spectrum of disorders includes motor dysfunction comprising oculomotor dysfunction [14]. More severe defects may manifest as infantile-onset epileptiform discharges over occipital brain regions. With disease progression, the seizures can generalize and can become resistant to medication. Other clinical features of severe forms of POLG-SDs include liver failure, cortical blindness, myoclonuses, and neurodegeneration [15].

POLG-spectrum disorders include a variety of syndromes, including Alpers–Huttenlocher syndrome (AHS), ataxia neuropathy spectrum (ANS), childhood myocerebrohepatopathy spectrum (MCHS), mitochondrial recessive ataxia syndrome (MIRAS), myoclonic epilepsy, myopathy sensory ataxia (MEMSA), and progressive external ophthalmoplegia (PEO) [16]. AHS is one of the most severe conditions among POLG-SDs. While early-onset (neonatal, infancy, or childhood) POLG-SDs such as MCHS or AHS are often associated with mtDNA depletion, the mtDNA defects in adolescent or young adult-onset disorders such as ANS, MIRAS, or PEO are mostly associated with (accumulating) mtDNA deletions [9]. Patients with POLG-SDs like AHS show decreased mtDNA content and increased mtDNA damage. The loss of mtDNA content (i.e., mtDNA depletion) can be tolerated to a certain extent without causing deleterious effects but is considered pathological when <70% [17]. MtDNA content is also correlated with the age of onset of POLG-SDs. Infants with POLG-SDs show less mtDNA content, with 80% loss, whereas adult patients show only 40–50% loss [17].

More than 300 pathological *POLG* variants have been described so far. The two most common mutations lead to the amino acid substitutions c.1399G>A; p.(Ala467Thr) and c.2243G>C; p.(Trp748Ser); patients with these mutations show significant residual (~5%) Pol-γ activity [15,18,19]. The c.1399G>A; p.(Ala467Thr) mutation alone is found in 36% of all alleles associated with POLG-SDs and exists in European populations with the carrier frequency of 0.2% to 1% [20,21]. Yet, there is a range of variants in the *POLG* gene that have been discovered and are suspected to be pathological [22,23]. Moreover, there are variants of unknown significance, indicating that it is unclear whether the variations have a significant effect on Pol-γ function.

To date, the *POLG*-variant c.678G>C; p.(Gln226His) has been described mostly as a variant of unknown significance in Clinvar (https://www.ncbi.nlm.nih.gov/clinvar/variation/206581/ accessed on 23 January 2025). The traditional CADD-score for the *POLG*-variant c.678G>C; p.(Gln226His) in its newest version (v1.7) is 24, thus >20, which is a hint towards pathogenicity. However, the current state-of-the-art prediction score Revel is 0.263, which is considered “neutral” (and thus not clearly benign but also not clearly damaging). Other bioinformatic predictions also result in a likely benign outcome. Thus, currently, there is not enough evidence to rate this variant as benign or pathogenic from a prediction/bioinformatic point of view. On the other hand, the c.2419C>T; p.(Arg807Cys) mutation is exclusively classified as pathogenic. In some cases, the compound heterozygosity of mutations in *POLG* can lead to a significant reduction in enzyme activity, which can even result in death during infancy, depending on the severity of the mutation, especially when a mutation is located in trans with another disease-causing mutation [16,24]. The compound heterozygosity of two *POLG* mutations may result in a more severe phenotype/shorter lifespan than the same mutations when present homozygously [25]. Since many patients diagnosed with a POLG-SD are compound heterozygous for two variants in *POLG* [15], the combination of different *POLG* mutations may strongly influence the phenotype of the patient [6].

## 2. Materials and Methods

### 2.1. Ethical Permission

The project was approved by the Ethics Commission of the Medical Faculty of the University of Cologne. Tissues were acquired with written informed consent.

### 2.2. Obtaining and Cultivating the Human-Derived Fibroblasts

Skin biopsies were collected from the patient and the carrier, and fibroblasts were isolated. Ethical approval was granted from the Ethics Commission of the Medical Faculty of the University of Cologne (13-022, 20 February 2019). Fibroblasts were cultured in a medium consisting of DMEM/F-12, GlutaMAX™ (Thermo Fisher Scientific, Karlsruhe, Germany, #10565-018), 10% (*v*/*v*) Fetal Bovin Serum (FBS, Biochrom AG, Schaffhausen, Schwitzerland), and Antibiotic-Antimycotic (1× Anti/Anti, #15240062, TFS). For maintenance, fibroblasts were cultivated in uncoated T75 cell culture flasks (VWR) in a sterile incubator (Heraeus HeraCell 150, Kendro, Heidelberg, Germany) at a temperature of 37 °C, 95% air humidity, and 5% CO_2_ concentration. The culture medium was changed once or twice per week, depending on the cell density, which was monitored, and images were taken with a phase-contrast microscope (DM IL LED, Leica, Wetzlar, Germany) using objectives with 10 and 20× (air-based) magnification (Leica) and Las X imaging software Version 3.7.6 (Leica). Cells were passaged for further cultivation or seeded for differentiation experiments at 70–80% confluency, and then these cells were washed with phosphate-buffered saline (PBS, TFS), trypsinized (0.05% Trypsin/EDTA in PBS, TFS) for 10 min, spun down at 1000 g for 5 min, and resuspended in the culture medium. For passaging, the culture was diluted 1:10 and seeded again in an uncoated T75 flask. For seeding, resuspended fibroblasts were counted with an automatic cell counter (TC20TM, Bio-Rad, Feldkirchen, Germany) and seeded with 3 × 104 cells/cm^2^. For long-term storage, trypsinized and spun-down cells were resuspended in FBS with 10% DMSO, cooled down with −1 °C/min in a cryo-container (Mr. Frosty^TM^, Thermo Fisher, Karlsruhe, Germany, VWR, Darmstadt, Germany), and stored at −80 °C or in liquid nitrogen.

### 2.3. Whole-Exome Sequencing and Annotation

The patient was previously reported on in a study published by Keller et al., 2021 [26]. Briefly, exome sequencing (ES) was performed as a parent–child duo experiment using the SureSelect All Exon kit (Agilent, Santa Clara, CA, USA) v7 following the Version D0, July 2018 protocol ’SureSelectXT Low Input Automated Target Enrichment for Illumina Paired-End Multiplexed Sequencing’. Libraries were prepared containing 10–200 ng genomic DNA for each sample. The process involved DNA fragmentation to 150–200 base-pairs, DNA end-modification (end-repair, dA-tailing, molecular-barcoded adaptor ligation), PCR amplification with index primers, and purification using AMPure XP beads. The prepared gDNA library, after quantity and quality assessment, underwent hybridization with the (target-specific) ‘SureSelect Capture Library’. The resulting hybrids were captured using magnetic beads with streptavidin coating, followed by PCR amplification and purification of the enriched DNA samples. Equimolar amounts of validated and quantified libraries were pooled. Quantification utilized the Peqlab KAPA Library Quantification Kit and Applied Biosystems 7900HT Sequence Detection System. Sequencing was performed on the Illumina NovaSeq6000 (San Diego, CA, USA) using a paired-end 100-nucleotide protocol, achieving an average 75× target coverage. For alignment, we used the hg38/GRCh38 human reference genome. Picard tools were used to remove PCR duplicates, while we used the Genome Analysis Toolkit (GATK) for local realignment and base quality score recalibration (BQSR). We called SNPs and short INDELs via Haplotype Caller, Platypus, and Mpileup programs, followed by variant quality score calibration (VQSR) using GATK. We used Conifer, XHMM, and ExomeDepth algorithms for coverage-based CNV detection. We used the ALLEGRO program for the detection of ROH through multipoint linkage analysis. Functional variant annotation and data combining were facilitated by the Cologne Center for Genomics (CCG)-developed modules FUNC and COMBINE.

### 2.4. Real-Time Quantitative PCR

DNA was extracted using a QIAGEN Mini DNA Kit (QIAGEN, Hilden, Germany) according to the manufacturer’s protocol. The quantification of mtDNA copy number was performed using RT-qPCR. The *MT-CYTB*, *MT-3319R* gene and the household genes *APP* and *18S* were amplified from all the fibroblasts using specific primers (see the next section). RNA was extracted using NucleoSpin RNA XS kit (MACHEREY-NAGEL, Düren, Germany) and cDNA was produced using the ProtoScript^®^ II First Strand cDNA Synthesis Kit (NEB, Ipswich, MA, USA). The quantification of mitochondrial-encoded mRNA levels was performed using RT-qPCR. The genes that were investigated were *MT-CYTB*, *MT-ATP6*, and *MT-ND1*, with *APP* as a household gene. Specific primers were used, flanking the named genes. RT-qPCR was performed with an initial denaturation step of 95 °C for 10 min then 95 °C denaturation for 15 s, followed by primer and probe hybridization and DNA synthesis at 60 °C for 60 s; the last two steps were repeated for 40 cycles. The reaction was measured using SYBR^®^ Green Reagent (Thermo Fisher) and the Applied Biosystems StepOnePlus (Thermo Fisher). The read-outs were analyzed with the ΔΔCT method.

### 2.5. Primers Used for RT-qPCR

APP Fw: TTTTTGTGTGCTCTCCCAGGTCTAPP Rev:TGGTCACTGGTTGGTTGGC18S rRNA Fw:TAGAGGGACAAGTGGCGTTC18S rRNA Rev:CGCTGAGCCAGTCAGTGTMT-CYTB Fw:GCCTGCCTGATCCTCCAAATMT-CYTB Rev:AAGGTAGCGGATGATTCAGCCMT-R3319 Fw: CACCCAAGAACAGGGTTTGTMT-R3319 Rev:TGGCCATGGGTATGTTGMT-ATP6 RT-qPCR Fw: CCCACTTCTTACCACAAGGCMT-ATP6 RT-qPCR Rev: TGGCGCTTCCAATTAGGTGCMT-ND1 RT-qPCR Fw: GCTTCAACATCGAATACGCCGCMT-ND1 RT-qPCR Rev: TAGAGTTCAGGGGAGAGTGCG

### 2.6. Fluorescence and Microscopy

Cells were cultivated as stated above in 24-well plates on glass coverslips (VWR), previously coated for 3 h at 37 °C with 20 µg/mL Poly-D-lysine (PDL, AppliChem, Darmstadt, Germany), fixed for 30 min at room temperature in 3.7% formaldehyde and 4% sucrose (in PBS) and permeabilized and blocked with 5% bovine serum albumin (BSA, Sigma-Aldrich, Saint Louis, MO, USA) and 0.2% Triton X-100 (AppliChem) in PBS for 10 min. Before fixation, cells were incubated with MitoTracker™ (Diulution 1:10,000) for 30 min at 37 °C and AlexaFluor™ Phalloidin (dilution 1:40) for 45 min at room temperature. Nuclei were stained with 1 drop/mL NucBlueTM (Hochest 33342, TFS) for 20–30 min, and samples were mounted on objective slides (Bio-Rad) with aqueous PolyMountTM (Polysciences, Warrington, PA, USA), dried for 24 at room temperature in the dark, and long-term stored at 4 °C in the dark.

Stained cells were imaged with a widefield fluorescence microscope (Axioscope 5, Zeiss, Oberkochen, Germany), using an LED excitation lamp (Colibri 7, Zeiss), a fluorescence camera device (Axiocam 705 mono, Zeiss), objective 40× (oil-based) magnification (Zeiss), ApoTome.2 (Zeiss) for pseudoconfocal image acquisition, and Zen imaging software (Zen 3.6 blue edition, Zeiss). For the analysis of protein expression levels, all images were taken with identical settings (laser intensity and exposure time) to ensure statistical comparability.

Images were analyzed with ImageJ 1.54f using a macro based on “Image J Mitophagy Macro” [27,28]. The macro calculated the relative area using the mitochondrial area (determined with MitoTracker™ Deep Red FM (Thermo Fisher)) and divided it by the Alexa Fluor™ 488 Phalloidin (Thermo Fisher) staining as a reference for the cell size. Interconnectivity was measured by dividing the mean area of the mitochondria by the perimeter of the mitochondria in the cell.

## 3. Results

### 3.1. Case Presentation

The patient, a 13-year-old male, first exhibited symptoms at 3 years of age. The initial symptoms were weakness and atrophy in the proximal muscles of the lower extremities and facial muscles. Over the years, the condition has involved the upper proximal extremities, voice, and back muscles, but has not shown progression afterwards. Physical examination revealed muscle atrophy, with more severe involvement of the lower limbs than the upper limbs. The patient displayed dysarthria, ptosis, scapular winging, pes cavus, and pes planovalgus. Muscle tone was notably decreased, and deep tendon reflexes were weakly elicitable on both sides. The patient reported significant fatigue and muscle pain following physical activity, although no sensory deficits were observed. Diagnostic tests including electromyography revealed no abnormalities, and the nerve conduction velocity was within normal limits. A muscle biopsy did not show any pathological changes. Imaging studies, including an MRI of the brain and spine, were normal, and serum creatine kinase levels were within normal range.

### 3.2. Experimental Results

**Figure 1:**
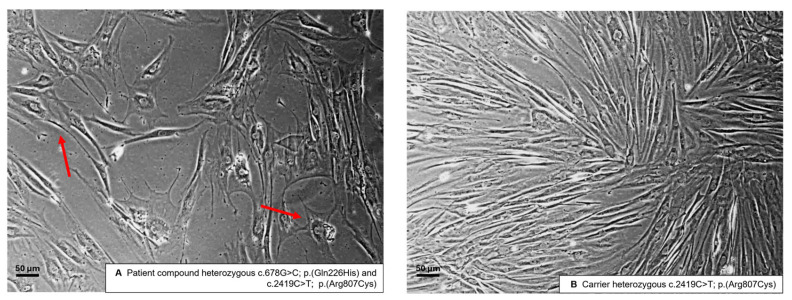
Phase-contrast microscopy images of fibroblasts (passage 4) from the patient and the relative show reduced growth in the patient cell line. The fibroblasts of the patient (**A**) are less confluent and have a polyclonal shape (examples indicated by red arrows). The fibroblasts of the carrier (**B**) grow faster, are more confluent, and have more of a linear shape. Scale bar: 50 μm.

To confirm a phenotype with mitochondrial impairment in vitro, we generated fibroblasts from skin biopsies derived from the patient (13-year-old male) and the mother (31-year-old female), as the only available first-degree relative. Both fibroblast lines were expandable, yet the patient’s cells grew slower than the cells of the carrier. Fibroblasts of the carrier show a more linear shape and appear less branched (Figure 1), with the patient’s fibroblasts showing sparser, less linear, and more diverse cellular morphologies, implying relative growth defects in an in vitro environment providing optimal conditions for fast cell proliferation.

### 3.3. c.678G>C; p.(Gln226His) Fibroblasts Have a Reduced mtDNA Content

**Figure 2:**
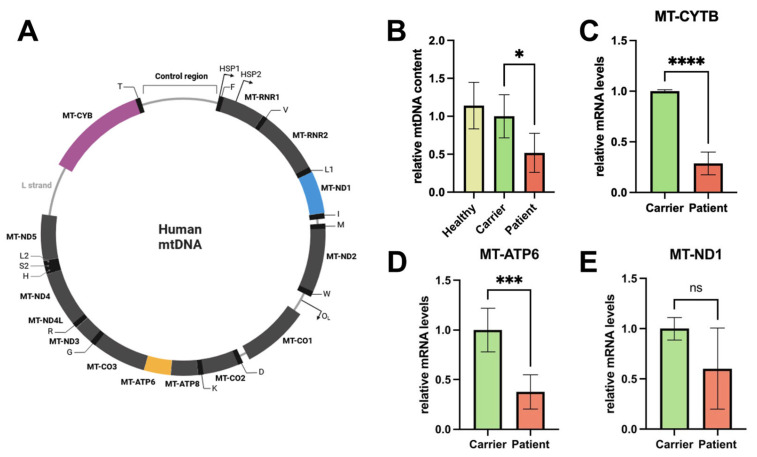
c.678G>C; p.(Gln226His) fibroblasts show reduced mtDNA levels and mtDNA-encoded mRNA levels as determined by RT-qPCR. (**A**) Scheme of the mitochondrial genome. It should be noted that MT-ND1 is closest to the origin of (heavy-strand) replication, while MT-CYTB is furthest away. (**B**–**E**): Fibroblasts of the patient and the relative were lysed, and DNA/RNA was isolated to perform RT-qPCR analysis. (**B**) The relative mtDNA content (calculated using genomic DNA and the nuclear gene APP as a reference) of the patient (red) and the carrier (green): reduction in mtDNA content ~50% (*n* = 3 (patient and carrier), *n* (healthy) = 4; carrier vs. patient; * *p*-value < 0.05 (0.0228); carrier vs. healthy *p*-value > 0.05 (0.5053)). (**C**–**E**) mRNA levels of the two cell lines of the mtDNA-encoded genes relative to the nuclear-encoded household gene APP. (**C**) *MT-CYTB*: mRNA levels are reduced by ~75% (*n* = 3; **** *p*-value < 0.0001). (**D**) *MT-ATP6*: mRNA levels are reduced by ~70% (*n* = 3; *** *p*-value > 0.001 (0.0004)).

Next, we used RT-qPCR to determine mtDNA content. We investigated the mitochondrial genes *MT-CYTB* and *MT-3319R*. For normalization, we used the household genes *APP* and *18S*. We found that the patient shows decreased mtDNA levels compared to the relative/carrier (a reduction of ~50%), indicating a severe loss of mtDNA replication due to the impaired function of Pol-γ in fibroblasts (Figure 2B). To ensure that the carrier had no lack of mtDNA content, we compared it to four controls with no *POLG* mutation and pooled them (1. female, 29 years old; 2. female, 15 years old; 3. young adult male; 4. male, 37 years old), showing that there was no significant difference between the carrier and the heathy controls.

### 3.4. c.678G>C; p.(Gln226His) Fibroblast mRNA Levels of mtDNA-Encoded Genes Are Reduced

Next, we investigated the effect of diminished mtDNA on mtDNA-encoded gene expression using RT-qPCR. To ensure that the whole circular mtDNA molecule was represented and to detect signs of the premature termination of transcription, we chose three genes located close to the transcription initiation of the heavy-strand promotor 2 (*HSP2*) (*MT-ND1*), in the middle of it (*MT-ATP6*), and in the end of the process (*MT-CYTB*). For normalization, we used *APP* as a household gene. Read-outs show that mRNA levels are affected by the malfunction of Pol-γ (Figure 2C–E), suggesting a reduction in mitochondrial function and fitness in these cells.

### 3.5. Mitochondrial Mass and Interconnectivity Are Negatively Affected by the Malfunction of Pol-γ

**Figure 3:**
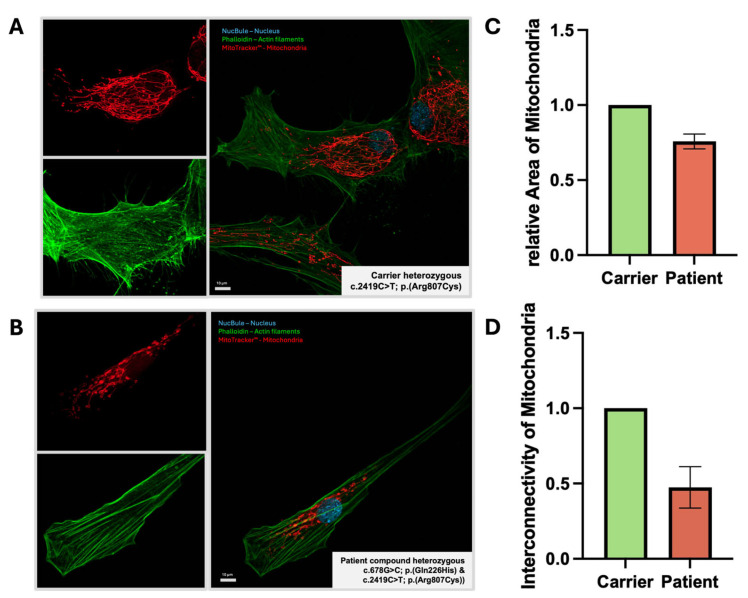
Fluorescent microscopy of fibroblasts, visualizing mitochondria with MitoTracker™, show reduced mitochondrial mass and interconnectivity in mitochondria. The fibroblasts were fixed and stained with MitoTracker™ (red color) to display the mitochondria and Phalloidin (green color) to recognize the cell size. (**A**) Representative image of the carrier fibroblast (upper panels) and (**B**) the patient (lower panels). (**C**) The relative area (also referred to as the mitochondrial mass) is measured by calculating the area of the mitochondria divided by the cell area (stained with CellTracker Phalloidin™): the relative area is reduced by ~20% (*n* = 2 of biological replicates, *n* = 10 of technical replicates). (**D**) The interconnectivity is calculated by dividing the mean area of the single mitochondria by the mean perimeter of one mitochondrion: the interconnectivity is reduced by ~50% (*n* = 2 of biological replicates, *n* = 10 of technical replicates). Scale bar: 10 μm.

To investigate changes in mitochondrial morphology, we stained mitochondria in fibroblasts with MitoTracker™. We visualized the relative area of mitochondria (the total area of the mitochondria divided by the total area of the cell) in individual cells and additionally calculated the interconnectivity (the mean area divided by the mean perimeter) (Figure 3). The relative area was reduced by ~20% (Figure 3C), and interconnectivity was reduced by ~50% (Figure 3D), in patient cells.

All in all, in our patient-derived fibroblasts, in comparison to a first-degree relative, we found that Pol-γ was likely impaired, as we found reduced mtDNA and lower mitochondrial mRNA and protein levels, all of which likely disrupted mitochondrial biogenesis, leading to decreased mitochondrial mass and interconnectivity.

## 4. Discussion

The present study aimed to investigate the impact of a specific variants in the *POLG*-gene c.678G>C; p.(Gln226His) on mitochondrial health by assessing mtDNA content, mRNA levels of mitochondrial genes, and mitochondrial mass and morphology in patient-derived fibroblasts of a patient carrying an additional and established heterozygous pathogenic variant in trans and showing clinical symptoms typical of mitochondriopathy. Our findings demonstrate a clear detrimental effect of the mutation on mitochondrial function and biogenesis in the studied cells in comparison to cells derived from a first-degree relative and are in agreement with the clinical manifestation as mitochondriopathy.

The experimental results show that compound heterozygous genetic variants in *POLG* c.678G>C; p.(Gln226His) and c.2419C>T; p.(Arg807Cys) result in impaired mitochondrial function. We found reduced mtDNA, mRNA levels of mtDNA-encoded genes, and mitochondrial area and interconnectivity. Our results show a significant reduction in mtDNA content in the mutant samples, i.e., the patient-derived fibroblast with the compound heterozygous genetic variants in *POLG* c.678G>C; p.(Gln226His) and c.2419C>T; p.(Arg807Cys), compared to the carrier control. This suggests that the heterozygous mutation c.678G>C; p.(Gln226His) in the *POLG*-gene impairs mitochondrial DNA replication when in trans with the established c.2419C>T; p.(Arg807Cys) mutation, in line with Pol-γ being the only mammalian enzyme responsible for mitochondrial DNA replication and with the patient showing a mitochondriopathy-typical phenotype. The observed decrease in mtDNA leads to the reduced expression of essential mitochondrial proteins (e.g., for OXPHOS), further compromising mitochondrial activity, such as energy production in forms of ATP in eukaryotic cells. Also, clinically, the patient phenotype would be in agreement with a reduction in mtDNA, with symptoms that can be caused by mitochondrial dysfunction (see below).

The mRNA levels of mitochondrial genes, including those involved in oxidative phosphorylation, were significantly lower in the patient’s cells. This reduction in mRNA levels indicates a disruption in the transcriptional regulation of mitochondrial genes, which could stem from the decreased mtDNA content naturally resulting in at least relatively decreased transcription in vitro in conditions of optimal cellular growth. The decreased transcription or premature termination of mtDNA in these genes alone is likely to impair the production of proteins necessary in mitochondrial respiratory function, leading to diminished ATP production, increased oxidative stress, and the impairment of crucial elements of mitochondrial biogenesis, such as mitochondrial fusion and fission [29]. It can be observed that the decreased proliferation capacity of the mitochondria results in attenuated fusion capacity, conversely provoking mtDNA depletion and mutation, as intermitochondrial mixing in heteroplasmic cells cannot complement pathogenic mtDNA, as demonstrated previously [29]. The effect of the impairment of Pol-γ probably has numerous downstream consequences (e.g., impaired mitochondria-targeted protein homeostasis, impaired oxidative phosphorylation, and impaired mitochondria-based metabolism), which could be investigated in further studies.

In the same way that the mtDNA content and mRNA levels of mitochondrial-encoded genes were reduced, our measurements of mitochondrial mass revealed a reduction in the patient samples. This finding indicates that the variant c.678G>C; p.(Gln226His), in conjunction with another pathogenic variant in trans, negatively affects not only the genetic and transcriptional components of mitochondria but also their overall abundance and structural integrity. This decrease in mitochondrial mass suggests a reduction in mitochondrial biogenesis and/or an increase in mitochondrial degradation. Moreover, the loss of mitochondrial mass will certainly lead to impaired cellular energy production, as fewer mitochondria are available to meet the metabolic demands of the cell.

Additionally, mitochondrial interconnectivity was significantly reduced. Mitochondrial interconnectivity refers to the dynamic network formed by mitochondria within a cell. This network facilitates efficient energy distribution, communication, and the exchange of mitochondrial DNA and proteins, ensuring optimal cellular function and adaptation to metabolic demands [30]. Mitochondrial interconnectivity is thus crucial in maintaining mitochondrial function and cellular homeostasis. The process of mitochondrial fusion is known to facilitate interconnectivity in human cells as it transfers gene products between mitochondria for optimal functioning, especially under metabolic and environmental stress [4]. Subsequently mitochondrial fusion might be involved in the ability of human cells to tolerate high levels of pathogenic mtDNA [29], caused by the impairment of Pol-γ, which replicates the mtDNA incorrectly. Therefore, mitochondrial fusion and its enhancement, as successfully shown in a former study [31], must be considered as a possible protective factor in the treatment of POLG-SDs.

However, while the read-outs appear valid, the conclusions that can be drawn are limited, as we only investigated the fibroblasts of one patient and a first-degree relative with the same mitochondrial DNA. While unlikely, it cannot be entirely ruled out that, for example, the two variants c.678G>C; p.(Gln226His) and c.2419C>T; p.(Arg807Cys) interfere with each other. This is unlikely, as the two variants are present in trans, and such an effect has not been demonstrated in association with *POLG*—however, this would not mean that the variant is not harmless but simply that it is pathogenic only in combination with the mutation c.2419C>T; p.(Arg807Cys). Also, as the fibroblasts investigated here have the same mitochondrial genome, it is not unreasonable to propose that the impaired mitochondrial function observed is due to impaired Pol-γ.

Age and sex differences in the assessed cell lines may influence the read-outs in the experiments. As former research proves that higher age correlates with lower mitochondrial function [32], the results shown in this study strengthen the hypothesis that reduced mitochondrial function in the patient’s (13-year-old) fibroblasts compared to the carrier’s fibroblasts (31 years old) may be pathological. Significant sex-dependent disparities in mitochondrial function have not been found in scientific research to date in humans, to the best of our knowledge.

### Clinical View and Conclusions

From a clinical perspective, patients with pathogenic variants in the *POLG*-gene affecting mitochondrial health may present a wide range of clinical symptoms, ranging from mild muscle weakness to stroke-like episodes and liver failure. The symptoms of our patient (e.g., muscle weakness and atrophy in different parts of the body and fatigue and muscle pain following exercise) are compatible with a mitochondriopathy. However, mitochondriopathy, in particular when caused by variants in *POLG*, can display a great variety of symptoms, meaning that it is difficult to argue that the patient presented here shows typical symptoms of a certain POLG-spectrum disorder, but his symptoms are, in principle, in agreement with a mitochondriopathy. Nonetheless, our study provides the first cellular evidence that the mutation c.678G>C; p.(Gln226His), when present in trans with a known pathogenic *POLG* variant, may well significantly impair mitochondrial health and function by reducing mtDNA content, mRNA levels of mitochondrial genes, and mitochondrial mass. These findings help explain the clinical symptoms observed in a patient with a mitochondrial disease. From a formal genetic perspective, the donor of the control fibroblasts, being the biological mother of the patient, shares a 50% identical genetic background and 100% of the mitochondrial genome, and also the second mutation present in the patient. This allowed us to investigate the c.678G>C; p.(Gln226His) variant independently of the other mutation present in the patient.

By comparing the heterozygous carrier (who shows no clinical symptoms) with the compound heterozygous patient (who exhibits clear and phenotypical symptoms), we can observe that it is possible that the c.678G>C; p.(Gln226His) variant of *POLG* is responsible for the patient’s condition. The observed detrimental effects, including reduced mtDNA content and mitochondrial mass, point to compromised mitochondrial function, and, in particular, impaired mtDNA replication. Future research should focus on unraveling the molecular mechanisms by which this variant disrupts mtDNA replication, maintenance, gene expression, and mitochondrial biogenesis. It is also important to determine whether the observed reduction in mitochondrial mass and the potential disruption of interconnectivity is a general response to mitochondrial dysfunction or specific to certain mutations, cell types, or conditions.

## Data Availability

Data will be provided upon reasonable request by the corresponding author.
